# Robotic-assisted early mobilization and virtual reality: a perspective on innovative support strategies for critically ill patients

**DOI:** 10.1186/s40635-023-00571-x

**Published:** 2023-12-06

**Authors:** Claudio Parco, Vanessa Kreuels, Malte Kelm, Christian Jung, Georg Wolff

**Affiliations:** 1 Department of Cardiology, Pulmonology, and Vascular Medicine, University Hospital Düsseldorf, Medical Faculty of the Heinrich Heine University Düsseldorf, Moorenstr. 5, 40225 Düsseldorf, Germany; 2 Cardiovascular Research Institute Düsseldorf, Medical Faculty and University Hospital Düsseldorf, CARID, Heinrich-Heine-University, Düsseldorf, Germany

**Keywords:** Virtual reality, Robotic-assisted early mobilization, Intensive care

**To the Editor**

Survivors of critical illness frequently suffer from physical, psychosocial and cognitive impairments which result in a substantial loss in quality of life. Many research groups are working on structured rehabilitation programs to reduce the burden and there are preventive approaches like early mobilization, which innovative technical solutions may help to integrate into daily practice: virtual reality (VR) devices [1] and robotic-assisted early mobilization offer new and promising opportunities to improve patient rehabilitation on intensive care units (ICU).

VR can be adapted to various applications, including clinical scenarios ranging from patient education and staff training to support of conscious sedation in invasive procedures [2, 3]. VR interventions have shown efficacy and minimal side effects, which makes them a potential supportive therapeutic option. However, quality of evidence on the influence of VR-based interventions in critically ill patients is limited and randomized controlled trials are lacking.

Early mobilization is an integral part of modern critical care, which aims to improve functional capacity of patients after discharge. However, timing of conventional early mobilization is challenging for both patients as well as ICU staff [4], as it may be hindered by tubes and lines necessary for ventilation, feeding and intravenous medication of the patient in the early stages of recovery and may even provoke hazardous situations potentially resulting in adverse events [5]. Robotic systems were thus developed to support early mobilization (e,g, VEMOTION®) consisting of a specialized bed and the actual robot facilitate verticalization and a subsequent walking experience. Since the patient is fastened to the robot in the ICU bed without the need to transfer the patient to a separate machine, hazards and necessary manpower are potentially reduced with this method.

We have introduced a combination of both VR and robotic-assisted devices to facilitate early mobilization (Fig. 1): We initially applied this combination to support recovery of a 57-year-old patient, who was treated at our ICU for septic pneumonia. He entered a phase of prolonged ventilator weaning after initial stabilization. He enjoyed this combination and asked to use it several times a day for up to one hour. There were no adverse safety events during his treatment. This initial experience encouraged of to apply this combination to selected patients that potentially benefit from both the physical and psychological effects during their prolonged ICU treatment. We have included additional information about the patients in the Additional file 1. There is however room for further refinement since synchronization of the robotic system and VR headsets was technically impossible.

**Fig. 1:**
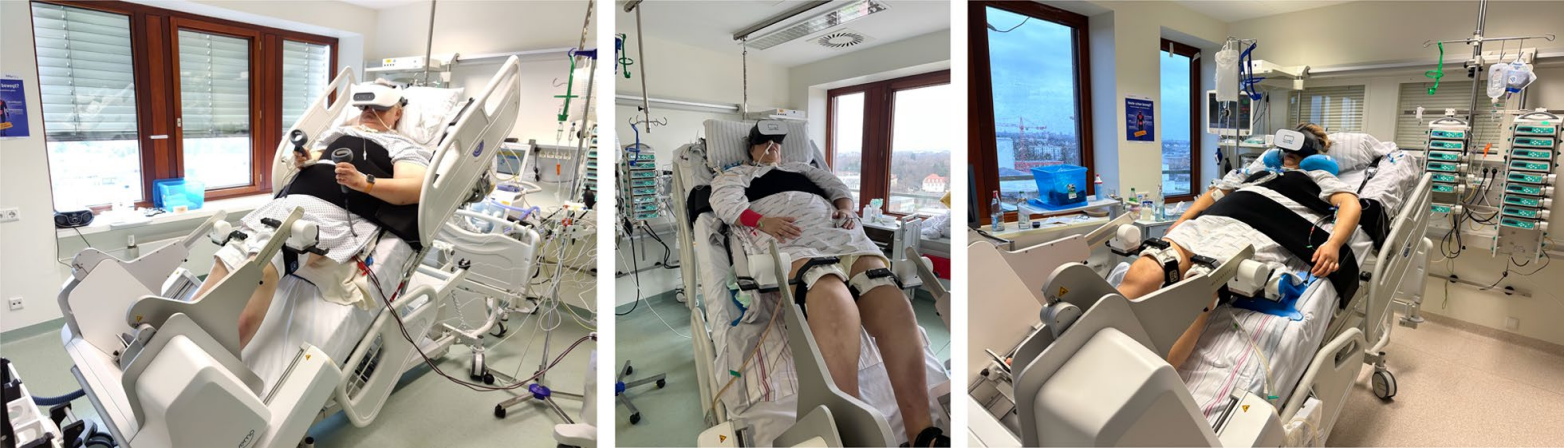
A combination of Virtual Reality (e.g. Oculus Meta Quest 2®) and robotic-assisted early mobilization (e.g. VEMOTION® system, Reactive Robotics) in three critically ill patients (for more information about the patients see Additional file 1)

## Conclusion

Prevention of physical and cognitive impairment following intensive care treatment requires innovative, multimodal therapeutic approaches: VR interventions and robotic-assisted early mobilization are easy to combine. However, there is a need for randomized clinical trials to further evaluate benefits and risks of these innovative methods while using standardized protocols and tools for outcome assessment. While application of this combination worked well in our selected patients, further research is necessary to prove feasibility in more challenging patients which e.g. suffer from delirium or motion sickness.

### Supplementary Information

**Additional file 1. **Background, patients and setting.

## Data Availability

Data sharing not applicable to this article as no datasets were generated or analyzed during the current study.
