# Supplementary material for: Robotic-assisted early mobilization and virtual reality: a perspective on innovative support strategies for critically ill patients
Source: Intensive Care Med Exp. 2023 Dec 6;11:86. doi: 10.1186/s40635-023-00571-x (PMC10697915; doi:10.1186/s40635-023-00571-x)
Supplement: Supplementary file 1 — Additional file 1. Background, patients and setting. [file 40635_2023_571_MOESM1_ESM.docx]

**Background, patients and setting**

**Virtual reality and robotic-assisted early mobilization in critical care medicine**

**Virtual and augmented reality**

Virtual reality (VR) and augmented reality (AR) are techniques which are commonly known from the entertainment industry. While with VR users are completely immersed into virtual three-dimensional environments, AR integrates virtual elements into a real-world environment. Both techniques offer the opportunity to assist both health care providers and patients.

From a physician or health care provider’s perspective, VR/AR can be used for various applications. Training of more complex procedures like priming of extracorporeal membrane oxygenation (ECMO) devices can be performed in a safe environment (1). Furthermore, especially augmented reality (AR) can be used to plan critical steps in interventional procedures (2). Besides planning and training of complex procedures, VR can be used to reduce stress in health care workers (3).

From a patient’s perspective, VR/AR offers the opportunity to support education (4) and recovery after critical care (5, 6). Moreover, VR/AR may be supportive options to reduce anxiety and discomfort (7) as well as pain (8). Previous work from our research group has summarized current literature about the use of VR/AR in critical care medicine (9-11).

**Robotic-assisted early mobilization**

Patients who survived critical illness frequently suffer from physical, psychosocial and cognitive impairments, which are often labeled as the “post-intensive care syndrome” (PICS) (12). The high prevalence of PICS results in a need for preventive approaches: Early mobilization is an integral part of a multimodal concept which aims to reduce the burden of PICS (13). Nevertheless, recent data from a randomized trial (TEAM) indicate that it is also associated with increased adverse events (14). Robotic-assisted early mobilization is a relatively new approach which aims to reduce staff resources involved in conventional early mobilization and to reduce side effects. However, until today, there are no data indicating any benefit over conventional early mobilization and robotic-assisted early mobilization devices are not yet integrated into daily clinical practice. The German project MobiStaR, which is funded by the Federal Ministry of Education and Research, currently evaluates the use of robotic-assisted early mobilization in intensive care medicine; there is also an ongoing single-centric feasibility study (15).

**Background information about patients and setting**

**Patients**

Figure 1 shows the application of a combination of virtual reality devices and robotic-assisted early mobilization in three patients which were treated at the medical intensive care unit of the University Hospital Düsseldorf.

Patient 1 as described in the letter was a 57-year-old man, who was in our critical care for septic pneumonia under immunosuppressive therapy after a heart transplantation in 2019. He deteriorated quickly in the acute phase of his illness and required mechanical ventilation and continuous renal replacement therapy, while he was provided guideline-recommended sepsis therapy. After initial stabilization, he entered a phase of prolonged ventilator weaning due to fluid accumulation and residual inflammation in his lungs. However, he soon communicated well and was motivated to participate actively in his recovery. We thus offered him both robotic-assisted mobilization as well as a VR-based simulation to support his recovery process. He was weaned off the ventilator successfully, however he later died unexpectedly due to a sudden respiratory event, unrelated to mobilization.

Patient 2 was a 45-year-old woman, who was treated for septic shock due to bilateral pneumonia. The patient’s condition worsened quickly leading to respiratory failure and need for mechanical ventilation. She was additionally diagnosed with hemophagocytic syndrome which was treated with chemotherapy. After initial stabilization, VR and robotic-assisted early mobilization were offered to her. She was motivated to use this combination and was discharged from the intensive care unit several weeks later after being weaned off the ventilator successfully and continued her recovery process on a normal care ward.

Patient 3 was a 46-year-old woman, who was treated due to a progressing paralysis because of myelitis, which resulted in tetraplegia and also affected auxiliary respiratory muscles. This soon led to respiratory failure and the need for mechanical ventilation. After initial stabilization, she was treated with plasmapheresis. Recovery was very slow, leading to a complicated and prolonged ventilator weaning. Despite being skeptical and anxious at first, she was willing to participate in our pilot study. Mobilization of patient 3 was more challenging than in the other two patients: Due to her neurological condition, she felt exhausted soon after we initiated mobilization which led to very short mobilization sessions of about 10 minutes at first. However, we were soon able to extend the sessions and she recovered well. She was discharged from the hospital around 3 months after the initial presentation.

**Preparation of early mobilization and human resources**

Before being allowed to use the system, the staff participated in a briefing given by the manufacturer in accordance with legal requirements. All patients were transferred to a special bed in which robotic-assisted early mobilization was possible. Those beds were provided by the manufacturer and could be used as normal ICU beds when robotic-assisted mobilization was not performed. The whole preparation process takes about 20 minutes depending on the experience of the involved personnel. Preparation of the mobilization therapy was performed by a physiotherapist together with an intensive care nurse. Personnel was guided through the preparation process step by step by the software of the robotic system: Patients’ legs and upper body were fixed to the robot using Velcro straps. Patients were then verticalized in their bed and mobilization was started with predefined parameters, using the embedded software. During mobilization, the physiotherapist took care of the patient and was able to adapt the range of motion and speed if necessary.

**Virtual reality (VR) devices**

Application of virtual reality was done using different devices (e. g. Oculus Meta Quest 2): Patients were able to choose from different immersive landscapes preinstalled on the VR headset. Patients were able to interact with the 3D landscape by turning their head in different directions. However, visual presentations were not in any way connected to the robotic system, therefore walking behaviors performed by the robotic system were not translated into the VR experience.

**Supplementary References:**

1. Wolff G, Bruno RR, Reiter M, Kantzow B, Kelm M, Jung C. Virtual reality device training for extracorporeal membrane oxygenation. Crit Care. 2020;24(1):390.

2. Chu MW, Moore J, Peters T, Bainbridge D, McCarty D, Guiraudon GM, et al. Augmented reality image guidance improves navigation for beating heart mitral valve repair. Innovations (Phila). 2012;7(4):274-81.

3. Nijland J, Veling W, Lestestuiver BP, Van Driel CMG. Virtual Reality Relaxation for Reducing Perceived Stress of Intensive Care Nurses During the COVID-19 Pandemic. Front Psychol. 2021;12:706527.

4. Zgoura P, Hettich D, Natzel J, Ozcan F, Kantzow B. Virtual Reality Simulation in Peritoneal Dialysis Training: The Beginning of a New Era. Blood Purif. 2019;47(1-3):265-9.

5. Vlake JH, Van Bommel J, Wils EJ, Korevaar TIM, Bienvenu OJ, Klijn E, et al. Virtual Reality to Improve Sequelae of the Postintensive Care Syndrome: A Multicenter, Randomized Controlled Feasibility Study. Crit Care Explor. 2021;3(9):e0538.

6. Vlake JH, van Bommel J, Hellemons ME, Wils EJ, Gommers D, van Genderen ME. Intensive Care Unit-Specific Virtual Reality for Psychological Recovery After ICU Treatment for COVID-19; A Brief Case Report. Front Med (Lausanne). 2020;7:629086.

7. Merliot-Gailhoustet L, Raimbert C, Garnier O, Carr J, De Jong A, Molinari N, et al. Discomfort improvement for critically ill patients using electronic relaxation devices: results of the cross-over randomized controlled trial E-CHOISIR (Electronic-CHOIce of a System for Intensive care Relaxation). Crit Care. 2022;26(1):263.

8. Rousseaux F, Dardenne N, Massion PB, Ledoux D, Bicego A, Donneau AF, et al. Virtual reality and hypnosis for anxiety and pain management in intensive care units: A prospective randomised trial among cardiac surgery patients. Eur J Anaesthesiol. 2022;39(1):58-66.

9. Kanschik D, Bruno RR, Wolff G, Kelm M, Jung C. Virtual and augmented reality in intensive care medicine: a systematic review. Ann Intensive Care. 2023;13(1):81.

10. Jung C, Wolff G, Wernly B, Bruno RR, Franz M, Schulze PC, et al. Virtual and Augmented Reality in Cardiovascular Care: State-of-the-Art and Future Perspectives. JACC Cardiovasc Imaging. 2022;15(3):519-32.

11. Bruno RR, Wolff G, Wernly B, Masyuk M, Piayda K, Leaver S, et al. Virtual and augmented reality in critical care medicine: the patient's, clinician's, and researcher's perspective. Crit Care. 2022;26(1):326.

12. Marra A, Pandharipande PP, Girard TD, Patel MB, Hughes CG, Jackson JC, et al. Co-Occurrence of Post-Intensive Care Syndrome Problems Among 406 Survivors of Critical Illness. Crit Care Med. 2018;46(9):1393-401.

13. Renner C, Jeitziner MM, Albert M, Brinkmann S, Diserens K, Dzialowski I, et al. Guideline on multimodal rehabilitation for patients with post-intensive care syndrome. Crit Care. 2023;27(1):301.

14. Group TSIatACT, Hodgson CL, Bailey M, Bellomo R, Brickell K, Broadley T, et al. Early Active Mobilization during Mechanical Ventilation in the ICU. N Engl J Med. 2022;387(19):1747-58.

15. Warmbein A, Schroeder I, Mehler-Klamt A, Rathgeber I, Huber J, Scharf C, et al. Robot-assisted early mobilization of intensive care patients: a feasibility study protocol. Pilot Feasibility Stud. 2022;8(1):236.
